# [^18^F]FDG PET/CT Radiomics for Predicting Pathological Risk Subtypes of Thymic Epithelial Tumors: A Bicentric Study

**DOI:** 10.3390/cancers18132038

**Published:** 2026-06-24

**Authors:** Antonio Sarubbi, Luca Frasca, Fatih Aksu, Guido Maria Meduri, Valerio Guarrasi, Gaetano Romano, Carmelina Cristina Zirafa, Filippo Longo, Gaetano Russo, Rosario Francesco Grasso, Paolo Soda, Franca Melfi, Pierfilippo Crucitti

**Affiliations:** 1 Department of Thoracic Surgery, Fondazione Policlinico Universitario Campus Bio-Medico, 00128 Rome, Italy; filippo.longo@policlinicocampus.it (F.L.); p.crucitti@policlinicocampus.it (P.C.); 2 Unit of Artificial Intelligence and Computer Systems, Department of Engineering, Università Campus Bio-Medico di Roma, 00128 Rome, Italy; fatih.aksu@unicampus.it (F.A.); valerio.guarrasi@unicampus.it (V.G.); p.soda@unicampus.it (P.S.); 3 Nuclear Medicine Unit, Fondazione Policlinico Universitario Campus Bio-Medico, 00128 Rome, Italy; guido.meduri@policlinicocampus.it; 4 Thoracic Surgery Unit, Cardiothoracic and Vascular Department, University Hospital of Pisa, 56124 Pisa, Italy; gaetano_romano1986@hotmail.com (G.R.); c.zirafa@gmail.com (C.C.Z.); 5 Unit of Diagnostic Imaging and Interventional Radiology, Fondazione Policlinico Universitario Campus Bio-Medico, 00128 Rome, Italy; gaetano.russo@unicampus.it (G.R.); r.grasso@policlinicocampus.it (R.F.G.); 6 Department of Diagnostics and Intervention, Radiation Physics, Biomedical Engineering, Umeå University, 901 87 Umeå, Sweden; 7 Unit of Thoracic Surgery, Department of Pharmacy and Health and Nutrition Sciences, University of Calabria, 87036 Rende, Italy; franca.melfi@unical.it

**Keywords:** machine learning, PET/CT, radiomics, thymic epithelial tumors, thymoma

## Abstract

**Background:** Thymic epithelial tumors (TETs) are rare mediastinal malignancies whose prognosis is largely determined by histology. Current predictive models rely on clinical variables and subjective imaging interpretation, with unsatisfied performance. Non-invasive pre-treatment risk stratification could guide surgical planning and perioperative management in patients with TETs. The role of fluorine-18 (^18^F) fluorodeoxyglucose (FDG) positron emission tomography/computed tomography (CT) in identifying aggressive disease is increasingly recognized. In this bicentric study, we aimed to evaluate a machine learning-based radiomics model using PET and CT images to differentiate between low-risk and high-risk TETs. **Methods:** Seventy-five patients who underwent PET/CT to evaluate the suspected anterior mediastinal mass and histopathologically diagnosed with TETs were included. On PET/CT images, the tumor was manually segmented by two experienced clinicians. First-order, shape, and texture features were extracted using the PyRadiomics library, resulting in 200 radiomics features (186 intensity/texture features and 14 shape features). In addition, rPET (i.e., tumor SUVmax/Liver SUVmax) parameter was included, yielding a grand total of 201 features. The feature set was reduced to 20 variables using ANOVA, with both selection and model evaluation performed via stratified 5-fold cross-validation. **Results:** The proposed approach achieved an average balanced accuracy of 0.58 ± 0.07 and an average AUC of 0.71 ± 0.04. Average sensitivity and specificity were 0.48 and 0.68, respectively. The model obtained an average Gmean of 0.57, indicating balanced and stable classification performance. **Conclusions:** Our ML models trained on PET/CT radiomic features showed moderate discriminatory performance for TET risk stratification.

## Simple Summary

Thymic epithelial tumors are rare thoracic cancers, and treatment decisions strongly depend on their aggressiveness. However, this information is usually confirmed only after surgery, while current imaging assessments may not always predict tumor behavior accurately. The authors aimed to develop and evaluate a machine learning model that uses positron emission tomography and computed tomography features to support non-invasive risk classification. Our preliminary findings suggest that image-based radiomic features may provide useful information about tumor aggressiveness.

## 1. Introduction

Thymic epithelial tumors (TETs) are rare neoplasms (0.15–0.32 cases per million in adults), but they account for up to half of anterior mediastinal masses [1].

The World Health Organization (WHO) histological classification is an independent prognostic factor, and it can be simplified into only three categories with significant survival differences: types A, AB and B1 = “low-risk” thymoma (LRT); B2 and B3 = “high-risk” thymoma (HRT); and thymic carcinoma (TC) in a separate class with poorer prognosis [2,3,4,5].

Despite its prognostic value, WHO histologic subtype is usually available after surgical resection [6,7]. Although needle biopsy may be essential in patients with suspected TETs, it is subject to sampling error, carries a risk of tract seeding and procedure-related complications [8]. Therefore, a reliable non-invasive estimation of the WHO classification via imaging studies might influence patient management in several ways.

Many features for predicting pathological and survival outcomes were proposed, but they often focused on clinical variables and subjective interpretation of images, with unsatisfied accuracy [9]. Conventional imaging, such as computed tomography (CT) and magnetic resonance imaging (MRI), is accurate to measure the extent of disease, although it has limited value for the prediction of histologic subtypes due to overlapping morphological features [10].

Over the past decade, fluorine-18 (^18^F) fluorodeoxyglucose (FDG) positron emission tomography/CT (PET/CT) has been found useful for assessing tumor invasiveness and for differentiating thymic hyperplasia from TETs [6,11,12]. The various metabolic patterns of the primary tumor can be measured on PET/CT through SUV-related parameters, such as maximum standardized uptake value (SUVmax) and volume-dependent parameters, such as metabolic tumor volume (MTV) and total lesion glycolysis (TLG) [13]. Nonetheless, these metabolic biomarkers demonstrate to predict TETs aggressive histopathology, disease recurrence, and survival, which may guide the management in terms of surgical plan and adjuvant therapy [8,13,14,15,16].

A meta-analysis revealed a significant difference in mean SUVmax between various risk classes of TETs; however, a clear cut-off value could not be identified [14]. Beyond SUVmax, MTV and TLG are not enough to distinguish between TET risk subtypes because of extensive overlap among them [17,18].

‘Radiomics’ is a computational approach characterized by the high-throughput extraction of numerous imaging features, converting medical images into high-dimensional data [19,20]. Machine learning (ML) algorithms can then be applied to these data to identify relevant imaging patterns and develop predictive models.

However, to our knowledge, the previous literature has strictly focused on single-modality radiomics, evaluating either PET or CT features in isolation. Investigating both modalities simultaneously is crucial, as they capture distinct but complementary biological phenomena: CT provides high-resolution structural and anatomical tissue density data, while PET captures functional metabolic variations.

In this context, our study provides the first bicentric evaluation of a dual-modality ([^18^F]FDG PET and CT) radiomics approach integrated with ML algorithms to classify TET malignancy grades. Given the rarity of these tumors and the challenges of multicenter data, this exploratory work focuses on exploring the feasibility and setting a methodological baseline for dual-modality feature extraction in TETs. Rather than presenting a finalized tool for direct clinical application, this study aims to evaluate the stability, boundaries, and current performance limitations of integrating structural and metabolic radiomics data.

## 2. Materials and Methods

### 2.1. Study Population

We conducted a retrospective analysis of records from patients with TET who were diagnosed after curative-intent surgical resection. We included patients treated between January 2020 to September 2025 at two large academic Italian hospitals devoted to mediastinal surgery: Fondazione Policlinico Universitario Campus Bio Medico of Rome (institution A) and Azienda Ospedaliero Universitaria Pisana of Pisa (institution B).

The inclusion criteria included the following: (1) [^18^F]FDG PET/CT imaging for evaluation of suspected anterior mediastinal mass within 4 weeks before surgery; (2) primary tumor with visible FDG uptake on the PET/CT reports; (3) ‘extended thymectomy’ performed via uniportal video-assisted or robotic thoracoscopic approaches (UVATS/RATS); (4) pathologically diagnosed TETs. Exclusion criteria were: (1) poor image quality; (2) history of prior resection for thymic neoplasm; (3) history of chemotherapy, or radiotherapy prior to the primary thoracic imaging; and (4) incomplete clinical, radiological, pathological and surgical data.

Following the selection criteria, a final cohort of 75 patients was included in the study. For the machine learning classification pipeline, patients were categorized into two primary risk groups based on histological subtyping: a Low-Risk Thymoma (LRT) group (n = 40) and a High-Risk group (n = 35). The High-Risk group comprised both high-risk thymomas (HRT) and thymic carcinomas.

This study was approved by the Institutional Ethics Committee (111.26 CET2 cbm, 11 June 2026). This study was conducted in accordance with the principles of the Declaration of Helsinki. Written informed consent to participate was obtained from all participants.

### 2.2. Definition and Data Collection

**Figure 1:**
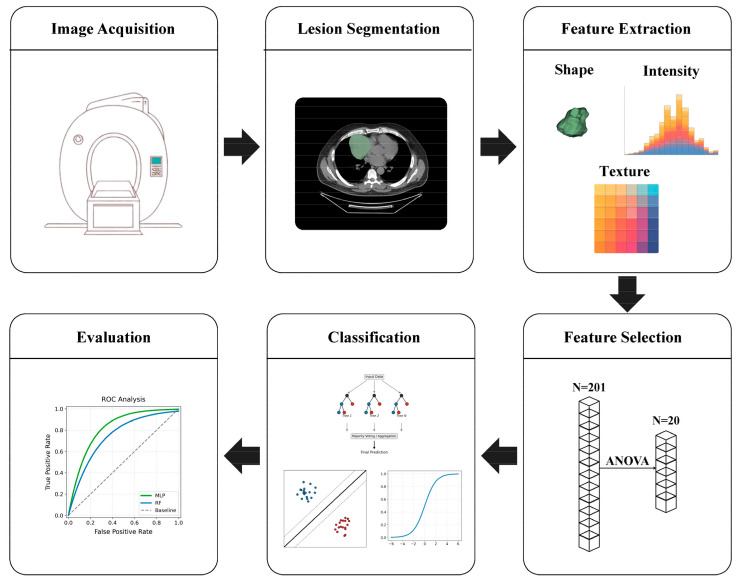
Overview of the radiomics workflow, illustrating the sequential stages of image acquisition, lesion segmentation, feature extraction and selection, and machine learning-based classification and evaluation.

TETs were classified as per the 2021 histological WHO classification [21]. According to aggressiveness and prognosis, all TETs were grouped into LRT (type A, AB and B1), HRT (type B2 and B3), and TC were included in the high-risk class, according to their poorer prognosis. Figure 1 outlines the radiomics workflow.

### 2.3. Image Acquisition and Tumor Regions of Interest Segmentation

Images were acquired according to standardized scan setup (FDG administration, acquisition timing, and TOF-PSF iterative reconstruction) in line with current European Association of Nuclear Medicine guidelines (EANM) recommendations, but different scanners and systems were used [Biograph Vision system (Siemens Healthineers, Erlangen, Germany) and Discovery VCT system (GE HealthCare, Chicago, IL, USA)] [22].

For each patient, images were reviewed by two board-certified radiologists with expertise in thoracic imaging, who were aware of the study’s purpose but were blinded to the clinical and pathological information.

Initially, co-localized CT images were evaluated using multiplanar reconstructions in the axial, sagittal, and coronal planes. Thymic lesion identification was guided by morphological and metabolic information, which were used to confirm lesion extent and to differentiate tumor tissue from surrounding mediastinal structures.

Tumor regions of interest (ROIs) were manually delineated on each axial enhanced CT slice using the open-source software 3D Slicer (version 5.8.1; www.slicer.org), with careful delineation of tumor contours on each slice. Manual CT-based segmentation was preferred over semi-automated PET threshold-based approaches to ensure complete lesion delineation and avoid potential underestimation of tumor extent, particularly in lesions with low or heterogeneous FDG uptake [15].

These ROIs were automatically combined by the software to generate three-dimensional volumes of interest (VOIs). The segmentation was subsequently refined on sagittal and coronal multiplanar reconstructions to encompass the entire visible primary lesion and exclude adjacent physiological FDG-avid structures [18]. Final segmentations were agreed upon by consensus. However, no independent double-segmentation experiment was performed, and inter-observer variability of the extracted radiomic features was not formally assessed.

From each VOI, we then extracted SUVmax, which indirectly estimated the maximum of FDG concentration within the VOI by the normalization with the patient’s body weight. An additional semiquantitative parameter, the rPET, was manually calculated using a fixed 2 cm^3^ region of interest positioned within liver parenchyma [7].

### 2.4. Feature Extraction and Selection

Radiomics features were extracted from both CT and PET images using the PyRadiomics library (v. 3.1.0) to quantitatively characterize the lesions [23]. A total of 201 features were computed, including 93 extracted from each modality. These comprised first-order statistical features characterizing the intensity distribution within the ROIs, along with various texture descriptors modeling spatial relationships and heterogeneity patterns. Specifically, texture features were derived from the gray-level co-occurrence matrix (GLCM), gray-level run-length matrix (GLRLM), gray-level size-zone matrix (GLSZM), neighboring gray-tone difference matrix (NGTDM), and gray-level dependence matrix (GLDM). Furthermore, 14 shape-based features were calculated to capture the geometric properties of the segmented lesions. Finally, the rPET parameter was incorporated into the set. This combined representation enabled a comprehensive characterization of both structural and metabolic lesion properties.

Prior to feature selection, Z-score normalization was applied to the features within each cross-validation fold to ensure a mean of zero and a unit variance. To reduce feature dimensionality and retain the most informative descriptors, a univariate feature selection strategy based on ANOVA was applied within a stratified 5-fold cross-validation (CV) framework. This approach was prioritized over more complex model-based selection techniques to ensure feature stability and minimize the risk of overfitting, which is critical given the high-dimensional nature of radiomic data relative to the available cohort size Features were then ranked based on their scores, and only the top ranked 20 features were selected for subsequent analysis, as this subset size was empirically determined to yield optimal model performance during sensitivity testing. This approach allows the identification of features that show the strongest association with the outcome while removing redundant and less informative variables.

### 2.5. Machine Learning Classifiers

Five ML models were evaluated for the classification of TET risk groups, namely a Multi-layer Perceptron (MLP), Logistic Regression (LR), Support Vector Machine (SVM), Random Forest (RF), and Gradient Boosting (GB), using the 20 selected features. All experiments were conducted under a 5-fold CV framework with stratification by the target variable and the institution to ensure a balanced and unbiased distribution of samples across training and validation sets. Feature selection was performed independently within each fold using only the training data, thereby preventing information leakage and ensuring a fair evaluation of model performance.

The MLP was implemented with two hidden layers consisting of 32 and 16 neurons, respectively, and Rectified Linear Unit (ReLU) activation functions were applied to each hidden layer. The model was optimized using the Adam optimizer with a learning rate of 0.001 and trained for 200 epochs. Following the ‘No Free Lunch’ theorem [24], all other models utilized default scikit-learn hyperparameters to provide a standardized baseline and prevent overfitting on the available sample size [25].

## 3. Results

**Figure 2:**
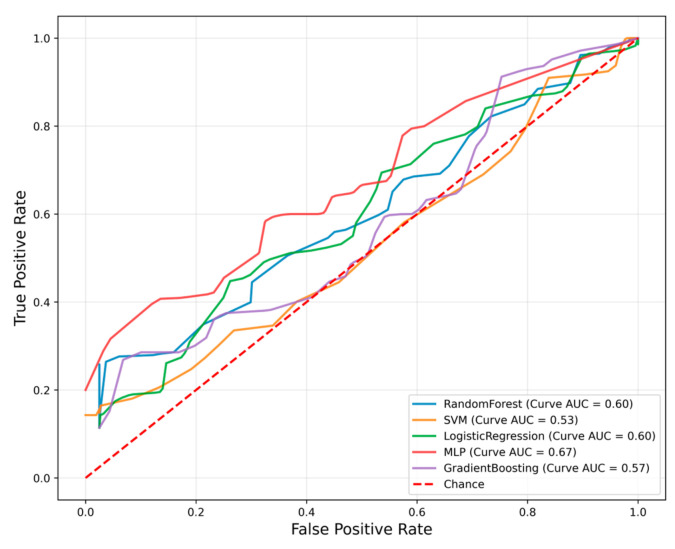
Receiver Operating Characteristic (ROC) curves for five machine learning models across 5-fold cross-validation. Curves illustrate the mean performance derived from threshold averaging across 5-fold cross-validation.

To evaluate classification performance, Receiver Operating Characteristic (ROC) curves were generated using threshold averaging across 5-fold CV experiments. Figure 2 illustrates the aggregated threshold performance across the cohort.

The Area Under the Curve (AUC) serves as the primary metric, while values reported in Table 1 represent the arithmetic mean of the five folds.

Among the five models, the MLP demonstrated the highest performance and stability, whereas the SVM performed near the baseline of chance with considerably higher variance. While RF and LR achieved identical mean AUCs, the latter exhibited a higher standard deviation. To assess differences in discriminatory performance, DeLong’s test was applied to the pooled out-of-fold predictions obtained from the five-fold cross-validation procedure. The results indicated lower *p*-values for comparisons between the MLP and the competing models, with the largest differences observed against SVM and GB (both *p* < 0.01). Similar trends were observed for RF and LR (both *p* < 0.05). Given that the predictions were derived from a cross-validation framework rather than a single independent test set, these *p* values should be interpreted as exploratory and supportive of the observed performance differences rather than as definitive measures of statistical significance.

Performance was further evaluated across several secondary metrics, including balanced accuracy (BACC), Geometric Mean (GMEAN), sensitivity (SEN), and specificity (SPE) (Table 1). The MLP model demonstrated the most consistent performance across these measures, achieving the highest scores in BACC and GMEAN which suggest a more stable equilibrium between classes compared to the other architectures.

While the MLP led in overall stability, other models exhibited specialized performance profiles. For instance, LR achieved the highest specificity, and RF yielded the highest mean sensitivity. However, these gains in specific areas often coincided with lower performance in the reciprocal metric. In contrast, the MLP’s results indicate a more balanced discriminative capacity. When considering the overall performance profile, combining the highest AUC, BACC, and GMEAN, the MLP emerges as the most robust architecture for this classification task.

**Figure 3:**
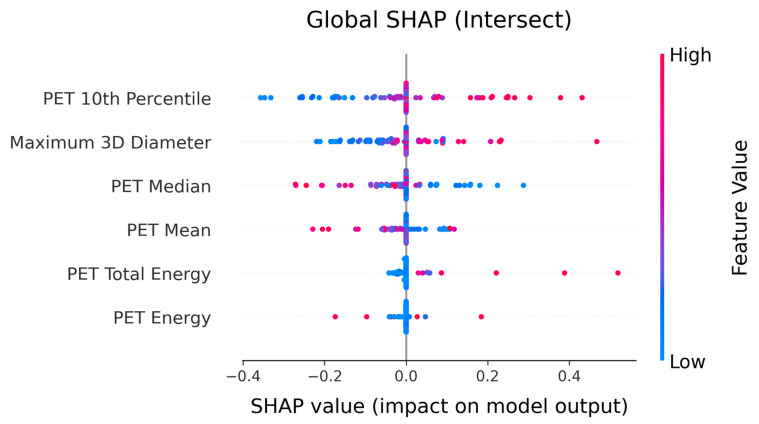
SHAP summary plot of the intersection of radiomic features consistently selected across 5-fold cross-validation for the MLP model, illustrating the global importance and directionality of influence. Feature values range from low (blue) to high (red), where a positive SHAP value (right of the center line) indicates an increased risk of high-risk TETs.

To further interpret the decision-making process of our top-performing model, we conducted an explainability analysis of the radiomic features selected during CV. Specifically, we examined the intersection of features that were consistently selected across all five folds to identify the most robust biomarkers for TET risk stratification. These common features were subsequently used to generate a SHAP summary plot (Figure 3), which illustrates both the global importance of each feature and the directionality of its influence on the model output [26].

The analysis demonstrated that the PET-derived 10th percentile intensity and the Maximum 3D Diameter were the most influential predictors, with higher values in both features being strongly associated with a high-risk TET classification. Interestingly, median and mean intensities followed these top predictors but exhibited an inverse relationship; once a high metabolic baseline was established by the 10th percentile, relatively lower average intensities pushed the model toward a high-risk prediction. We hypothesize that this configuration could reflect a relatively homogeneous ‘plateau’ of metabolic activity within aggressive tumors; however, this remains an exploratory post hoc observation requiring independent biological validation.

To further explore the interpretability of these multivariate findings, we performed a complementary univariate statistical analysis on this robust feature set. The detailed performance metrics and clinical thresholds for the six robust features identified through the intersection of the CV folds are summarized in Table 2.

Univariate comparisons were performed using the Mann–Whitney U test. The results confirmed that the PET-derived 10th percentile intensity was the strongest standalone biomarker, demonstrating a statistically significant difference between risk groups (*p* = 0.019, AUC = 0.66). Using Youden’s index, we identified an optimal clinical threshold for this feature at 1.24, identifying a specific cut-off for high-risk stratification. While other features such as Maximum 3D Diameter (*p* = 0.075, AUC = 0.62) and Mean Intensity (*p* = 0.092, AUC = 0.61) showed notable trends toward risk separation, they did not achieve independent statistical significance at the α = 0.05 level. The fact that the integrated MLP model achieved a superior AUC (0.71) compared to any single feature underscores the synergistic value of the multivariate approach. By moving beyond simple thresholds to capture the complex interplay between the metabolic ‘floor’ (10th percentile) and tumor dimensions, the machine learning model provides a more nuanced and accurate assessment of thymic epithelial tumor aggressiveness.

**Figure 4:**
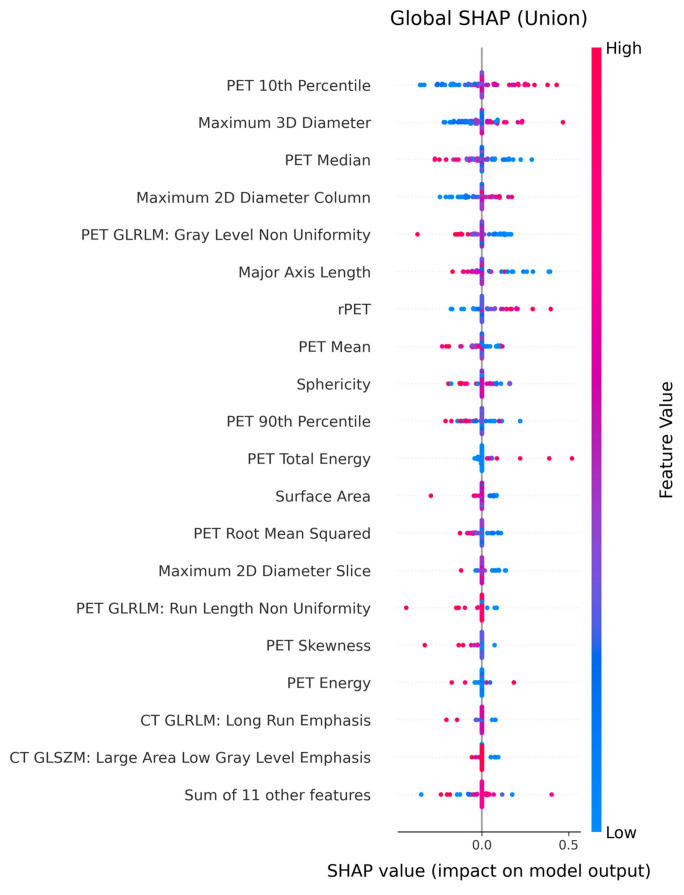
SHAP summary plot representing the union of all radiomic features selected across 5-fold cross-validation for the MLP model, demonstrating the global importance and predictive influence of the comprehensive set of biomarkers identified during TET risk stratification. Feature values range from low (blue) to high (red), where a positive SHAP value (right of the center line) indicates an increased risk of high-risk TETs.

To further analyze the selected features across folds, we generated another SHAP plot representing the union of all features selected across the five folds (Figure 4).

This aggregated view provides a wider picture of the model’s information sources and confirms the overwhelming dominance of PET-derived metrics in the risk-stratification process. While the top three features—10th percentile intensity, Maximum 3D Diameter, and Median intensity—remained consistent with the intersection analysis, the union revealed secondary markers that contribute to the model’s predictive power. Specifically, texture features such as Gray Level Non-Uniformity (GLRLM) and clinical ratios like the rPET appeared in the union, suggesting they capture auxiliary signals related to tumor heterogeneity and relative metabolic burden. Furthermore, the plot highlights the secondary role of CT features, such as Long Run Emphasis and Large Area Low Gray Level Emphasis, which appear only at the lower end of the importance spectrum.

## 4. Discussion

The present study aimed to evaluate the feasibility of a ML-based radiomics model derived from preoperative [^18^F]FDG PET/CT images for the non-invasive stratification of TETs according to WHO risk categories. Using a rigorous CV framework, our model achieved a maximum AUC of 0.71 with the MLP, associated with a balanced accuracy of 0.58, sensitivity of 0.48, and specificity of 0.68, indicating a moderate but reproducible discriminative performance.

Most previous PET-radiomics studies have focused on textural heterogeneity features in discriminating TET subgroups, often reporting higher AUC values but limited interpretability [9,27]. Lee et al. suggested a complementary role for textural heterogeneity and peak metabolic activity [8], while Nakajo and colleagues demonstrated that combining SUVmax with selected texture parameters improved diagnostic performance [28]. However, in our analysis, textural parameters were not consistently retained across folds. While features like GLRLM emerged as valuable secondary descriptors in the aggregated union (Figure 4), they lacked the fold-to-fold stability of first-order metrics.

Consistent with literature, SUVmax remains a central surrogate metabolic marker in TET characterization, reflecting tumor aggressiveness and metabolic activity [14]. While SUVmax has been shown to be particularly useful for distinguishing TC from LRT, the substantial overlap among TET subgroups precludes the identification of a reliable SUVmax cut-off value [7,8]. Importantly, our analysis highlighted that SUVmax alone does not drive model predictions. Instead, PET-derived 10th percentile intensity (*p* = 0.019, AUC = 0.66) emerged as the most influential predictor. As a hypothesis-generating finding, this pattern may suggest that high-risk tumors tend to exhibit a ‘plateau-like’ distribution, where a large portion of the tumor volume maintains a uniformly high metabolic ‘floor.’ Further histopathological and prospective imaging studies are needed to determine the true underlying physiological mechanism.

Crucially, the model’s performance relies on the interplay between this metabolic signature and structural context. Although Maximum 3D Diameter lacked univariate significance (*p* = 0.075, AUC = 0.62), it served as a “volume-multiplier” for metabolic signals within the multivariate framework. By integrating dimensions with intensity, the model moves beyond morphology-dominant approaches to provide a more comprehensive representation of tumor behavior [6].

Furthermore, we investigated the added value of the rPET ratio, which was identified as the seventh most influential predictor; univariate analysis confirmed that high-risk tumors had significantly higher values (2.80 ± 3.55 vs. 1.57 ± 0.76, *p* = 0.018) [7,29,30]. With a standalone AUC of 0.667, the optimal clinical threshold for rPET was determined to be 1.84, suggesting that a tumor-to-liver uptake ratio exceeding this value serves as a strong indicator of high-risk TET pathology. Therefore, rPET significantly contributed to the overall interpretability of the model [7].

From a clinical perspective, radiomics has consistently demonstrated strong potential for TET risk stratification, offering valuable insights into tumor aggressiveness and supporting personalized treatment planning [31]. PET-derived metabolic radiomics, integrating structural and functional tumor features, may improve non-invasive assessment in selected or borderline cases, where unexpected TET diagnoses after thymectomy for presumed thymic hyperplasia or benign conditions underscore the limitations of conventional preoperative evaluation [6,11]. In current clinical practice, resectable thymic tumors are often managed with upfront surgery without prior histological confirmation. For patients with LRT, complete surgical resection is generally considered curative and represents the primary therapeutic approach when technically feasible. In contrast, patients with HRT or TC often require a multimodality treatment strategy combining surgery with adjuvant chemotherapy and/or radiotherapy [27]. Accurate preoperative stratification may also influence surgical planning in patients with TETs, guiding the choice between thymomectomy and extended thymectomy, based on tumor biology and the likelihood of achieving complete resection [32]. In this context, PET/CT-based radiomic models may further reinforce the indication for lymph node sampling in high-risk TETs, justifying a more selective nodal approach in LRT.

Despite these promising results, several limitations remain. First, the sample size of 75 patients is relatively small for a radiomics pipeline extracting 201 initial features, which inherently elevates the risk of overfitting. To mitigate this high feature-to-sample ratio, we implemented strict computational guardrails, including isolated intra-fold feature reduction down to 20 features and an out-of-fold 5-fold cross-validation framework to prevent data leakage. Second, due to the lack of an independent external validation cohort, all reported performance metrics represent internal validation trends only, which limits the immediate generalizability of our findings. Consequently, our results should be interpreted as a preliminary, hypothesis-generating framework. Additionally, the retrospective design, the heterogeneity in study methodologies and the variability in segmentation approaches further affect reproducibility. Of note, although segmentations were finalized by consensus, inter-observer variability and Intraclass Correlation Coefficients (ICC)-based radiomic feature stability were not formally assessed. Furthermore, the use of two different PET/CT scanners may have introduced scanner-related variability in radiomic feature extraction, as no formal harmonization procedure (e.g., ComBat correction) was applied.

Finally, development of deep learning (DL) architectures is required to improve model accuracy and robustness [33,34].

## 5. Conclusions

In this exploratory study, ^18^F-FDG PET/CT-based radiomic analysis using a ML approach showed moderate performance for the preoperative prediction of pathological risk subtypes in TETs.

Larger multicenter studies with external validation are needed to deepen the understanding of the relationship between histologic features, clinical data, and medical images and to assess the role of radiomics in clinical practice to assist clinicians in treatment decisions.

## Figures and Tables

**Table 1:** Performance metrics of ML models for TET risk stratification across 5-fold cross-validation; values are presented as Mean ± Standard Deviation for AUC, BACC, GMEAN, SEN and SPE.

**Model**	**AUC**	**BACC**	**GMEAN**	**SEN**	**SPE**
MLP	0.71 ± 0.04	0.58 ± 0.07	0.57 ± 0.08	0.48 ± 0.12	0.68 ± 0.08
RF	0.61 ± 0.10	0.55 ± 0.07	0.54 ± 0.07	0.54 ± 0.07	0.56 ± 0.11
LR	0.61 ± 0.13	0.56 ± 0.08	0.52 ± 0.11	0.42 ± 0.17	0.70 ± 0.15
GB	0.56 ± 0.10	0.45 ± 0.10	0.45 ± 0.09	0.48 ± 0.11	0.43 ± 0.10
SVM	0.51 ± 0.18	0.57 ± 0.06	0.51 ± 0.10	0.37 ± 0.15	0.65 ± 0.14

Area Under the Curve (AUC), balanced accuracy (BACC), Geometric Mean (G-Mean), sensitivity (SEN), and specificity (SPE).

**Table 2:** Statistical comparison and univariate diagnostic performance of the top-ranked radiomic features for differentiating low-risk and high-risk TETs; results include the Mean ± Standard Deviation for each risk group, *p*-values from comparative testing, and univariate Area Under the Curve (AUC) with corresponding optimal thresholds.

**Feature**	**Low-Risk (Mean ± Std)**	**High-Risk (Mean ± Std)**	***p*** **-Value**	**AUC**	**Threshold**
10th Percentile	0.91 ± 0.37	1.17 ± 0.54	0.019	0.66	>1.24
Maximum 3D Diameter	54.87 ± 18.23	68.92 ± 31.44	0.075	0.62	>81.35
Median	1.74 ± 0.68	2.42 ± 1.50	0.097	0.61	>2.43
Mean	1.82 ± 0.68	2.52 ± 1.70	0.092	0.61	>2.42
Total Energy	1.61 × 10^5^ ± 2.65 × 10^5^	10.47 × 10^5^ ± 22.50 × 10^5^	0.224	0.58	>5.17 × 10^5^
Energy	0.50 × 10^5^ ± 0.86 × 10^5^	3.81 × 10^5^ ± 9.39 × 10^5^	0.331	0.57	>1.20 × 10^5^

Area Under the Curve (AUC).

## Data Availability

The data presented in this study are available on request from the corresponding author due to privacy.

## Abbreviations

The following abbreviations are used in this manuscript:

TETs	Thymic epithelial tumors
WHO	World Health Organization
LRT	Low-risk thymoma
HRT	High-risk thymoma
TC	Thymic carcinoma
CT	Computed tomography
MRI	Magnetic resonance imaging
18F	Fluorine-18
FDG	Fluorodeoxyglucose
SUVmax	Maximum Standardized Uptake Value
MTV	Metabolic Tumor Volume
TLG	Total Lesion Glycolysis
ML	Machine Learning
UVATS	Uniportal Video-Assisted Thoracoscopic Surgery
RATS	Robotic-Assisted Thoracoscopic Surgery
ROIs	Tumor Regions of Interest
VOIs	Volumes of Interest
GLCM	Gray-level co-occurrence matrix
GLRLM	Gray-level run-length matrix
GLSZM	Gray-level size-zone matrix
NGTDM	Neighboring gray-tone difference matrix
GLDM	Gray-level dependence matrix
CV	Cross-validation
MLP	Multi-layer Perceptron
LR	Logistic Regression
SVM	Support Vector Machine
RF	Random Forest
GB	Gradient Boosting
ROC	Receiver Operating Characteristic
AUC	Area Under the Curve
BACC	Balanced Accuracy
GMEAN	Geometric Mean
SEN	Sensitivity
SPE	Specificity
DL	Deep-Learning
